# Do health care providers give sufficient information and good counseling during ante-natal care in Lao PDR?: an observational study

**DOI:** 10.1186/s12913-019-4258-z

**Published:** 2019-07-04

**Authors:** Sysavanh Phommachanh, Dirk R. Essink, E. Pamela Wright, Jacqueline E. W. Broerse, Mayfong Mayxay

**Affiliations:** 1 Institute of Research and Education Development, University of Health Sciences, Ministry of Health, Samsenthai Street, Pearvath Village, Sisathanark District, P.O. Box: 7444, Vientiane Capital, Lao PDR; 2 Athena Institute and Amsterdam Public Health Institute, Vrije Universiteit Amsterdam, Amsterdam, The Netherlands; 3 Guelph International Health Consulting, Guelph, Ontario Canada; 4 Lao-Oxford-Mahosot Hospital-Wellcome Trust Research Unit (LOMWRU), Microbiology Laboratory, Mahosot Hospital, Vientiane, Lao PDR; 5 Centre for Tropical Medicine and Global Health, Nuffield Department of Clinical Medicine, University of Oxford, Oxford, UK

**Keywords:** Health education, Counseling, Public ANC services, Urban-rural areas, Laos

## Abstract

**Background:**

It is increasingly recognized that improving the quality of maternal health care delivery is of utmost importance in many countries. In Laos, the quality of antenatal care (ANC) service remains inadequate, but it has never been assessed thoroughly. This study aims to determine the ANC quality at the urban and rural public health facilities in Laos and provides suggestions to improve health education and counseling in addition to other routine care in public ANC services.

**Methods:**

This health-facility based, cross-sectional observation study included both health providers (*n* = 77) and pregnant women (*n* = 421) from purposively selected health facilities (*n* = 16). Information on the mothers’ current pregnancies, previous visits and their last children was collected. The time spent for each ANC session as well as ANC services provided were recorded. Descriptive and inferential statistics were applied to analyze the data.

**Results:**

Overall performance of ANC services by health care providers was poor in both urban and rural areas. Insufficient provision of information on danger signs during pregnancy, nutrition, breast feeding and iron supplements was revealed. Generally the communication skills, behavior and attitude of health providers were very poor. Less than a quarter of pregnant women were treated with kindness and respect. Only 4% of the observed ANC session took privacy into consideration. Less than 10% of available information materials were used during each ANC session. None of the health providers in both rural and urban areas performed specific counseling. Overall mean (SD) time-spent for each ANC session was 16.21 (4.28) minutes. A positive correlation was identified between the length of working experience of health providers and their physical performance scores (adjusted R square = 0.017).

**Conclusions:**

The overall performance of ANC services by health care providers was inadequate in both urban and rural areas. Insufficient provision of health education and poor communication skills of health care providers were revealed. Existing IEC materials were scarcely used. Taking action to improve the quality of ANC services by training and providing specific guidelines, creating dedicated rooms, and providing sufficient and effective materials for counseling are all greatly needed in public health facilities in Laos.

## Background

Effective pre-, intra-, and post-natal care interventions play an important role in reducing maternal and child mortality and morbidity [1–5]. Antenatal care (ANC) is the care that a woman receives throughout her pregnancy and for some weeks during the post-partum period in addition to identification of problems occurring during pregnancy [1, 6], ANC provides an excellent opportunity to establish a birth plan and to promote a healthy lifestyle that improves health outcomes for the woman, her unborn child and possibly her family [7, 8]. Besides medical history-taking and physical examination, treatment and referral to a hospital, health education and counseling play an important role in ANC [9–11].

Good health education and counseling in ANC means that a pregnant woman should be fully informed about the progress of her pregnancy and that she is provided with evidence-based information and support to make informed decisions, in a manner suitable to her culture and life experience [10, 12]. Pregnant women and their family members should have the opportunity to be informed about care, treatment, and possibility of unexpected matters, in partnership with their health care providers [12].

A previous study demonstrated that more women changed their behavior for reducing adverse pregnancy outcomes after having received nutrition counseling [13, 14]. In other studies, the positive effect of health education and counseling was significantly associated with improvement of maternal behaviors and health outcomes [13, 15, 16]. It has been shown that individual counseling with weekly reinforcement can bring about improvement in nutritional status during pregnancy [17].

However, in actual practice, the quality of ANC services is often poor in low-and middle-income countries and the information provided often does not match the social and cultural context of women to promote their healthy behavior at home [12, 18]. Improving this approach will strengthen the link between women and their family members and the health services during and after childbirth and may also help to promote delivery at health facilities, breastfeeding and a healthy lifestyle [12, 18].

Laos, where this study was done, is no exception. Although access to ANC has improved over the past decade [19, 20], the quality of ANC service remains inadequate [21], and there are indications that especially counseling is usually not practiced well at public health facilities [22]. The poor health education and counseling provided by health care workers has not yet been explicitly addressed in health interventions, just as poor public awareness and empowering women have not.

Very little is known about the current practice of Lao health care workers providing ANC services at the public health facilities. A recent study provided evidence of the perspectives and experiences of both supply and demand sides as to this issue, based on in-depth interviews (submitted for publication). However, the actual providers’ practice on health education and counseling was not described in that report. To address this knowledge gap, we conducted an observational study on the current practice of ANC health education and counseling to pregnant women by health providers at public health facilities in both urban and rural areas. The study aims to assess the ANC quality at the public health facilities in Laos; the results provide suggestions to improve health education and counseling in addition to other routine care in public ANC services.

## Methods

### Study design and sites

We conducted a health facility based, cross-sectional observational study, covering 16 public health facilities that routinely provide ANC services. The four central hospitals (Mahosot, Setthathirath, Mother and Newborn, and Friendship) in Vientiane Capital are located in urban settings, while the two provincial hospitals (Salavan and Attapeu), four district hospitals (two in Salavan and two in Attapeu), and eight health centers (four in Salavan and four in Attapeu) are located in rural areas.

Classification of urban and rural areas is based on the distribution of the population [23]. The population density of Salavan and Attapeu provinces is less than 50 people per Km^2^ while that of Vientiane Capital is 209 per Km^2^ [24].

These health facilities were purposively selected for the study for the following reasons: (1) these health facilities represent both urban and rural areas, as well as different levels (central, provincial, district hospitals and local health centres); (2) these facilities were also used in a previous study using in-depth interviews to explore the perspectives and experiences of supply and demand sides on the quality of ANC provision by health care providers, aimed at improving quality of ANC provision (submitted for publication), so that conducting observation to assess the quality of ANC in the same areas helps to relate the information from the real practice of health care providers to compare with the previous results. Perhaps most importantly, we plan to use the information from both interviews and the current observational study as a baseline for future interventions in these areas.

The sample size for the study was calculated using http://www.calculator.net/sample-size-calculator.html and based on the available Lao data from 2016, that the number of households with a pregnant woman is 192,000 and the Lao Social Indicator Survey in 2017 showing that 87.4% of Lao pregnant women attended at least one ANC visit [20]. We should recruit at least 420 pregnant women and we recruited 421 (81 were from urban and 340 from rural areas as shown in Table 1). Distribution of number of participants to be observed for each ANC session in the urban and rural areas was based on the feasible number of observations per day and all health providers who provided ANC service were included.

**Table 1:** Distribution of numbers of participants in the urban and rural areas

Estimated number of observation per day	Working day	Total estimation of observation	Final number of pregnant women	Number of non consent	Number of Health providers	Health facilities	Urban and rural areas
6	5	30	20	10	4	Central level1 (Mahosot)	Urban area
6	5	30	21	9	4	Central level2 (Sethathirath)	
6	5	30	20	10	5	Central level3 (Mother&Newborne)	
6	5	30	20	10	3	Central level4 (Friendship)	
		120	81	39	16		
8	5	40	40	0	4	Attapeu province	Rural area
7	5	35	35	0	3	Saysetha district	
7	5	35	35	0	3	Xanxay district	
7	5	35	33	2	3	Sanamxay district	
7	5	35	33	2	3	Phouvong district	
		180	176	4	16		
8	5	40	40	0	4	Salavane province	
7	5	35	32	3	3	Vapy district	
7	5	35	31	4	3	Laognam district	
7	5	35	30	5	3	Khongxedonh district	
7	5	35	31	4	3	Toumlane district	
		180	164	16	16		
Grand total	Sub total	480	421		48		

### Study participants

This study involved both health providers and pregnant women who attended ANC. Health providers including doctors, nurses, and midwives currently practicing at the ANC services with at least 1 year of working experience at that health facility were invited to participate. Pregnant women and family members who visited the study site ANCs during the observation sessions were also invited.

### Research instruments

A checklist for ANC session observation was developed from “The WHO recommendation on ANC services for positive pregnancy experience” and a counseling handbook of WHO [10, 11], both of which are used to train Lao ANC providers. The checklist had five sections: (1) medical history taking; (2) physical examination; (3) providing health promotion during pregnancy, for childbirth and postpartum care; (4) health education material to be used during ANC provision; and (5) effective communication skills, behavior and attitude of health providers. The checklist was firstly developed in English; thereafter back and forth translation was made between English and Lao languages to ensure accuracy. The checklist tool in Lao language was pilot tested at different health facilities in the study areas and edited where needed. The checklist can be provided as requested.

### Data collection

Data collection was carried out from July to August 2017. Two Lao nurses and two midwives with two to 4 years’ working experience in ANC from Vientiane were recruited and trained for data collection. Authors SP and MM trained them for 3 days, using role-play to demonstrate how to observe the performance of the health providers and fill in the observation checklist. During the training, the four data collectors practiced data collection at the ANC of health facilities not included in the study. They were closely monitored and supervised in the field by the two trainers.

Prior to data collection, the health providers and pregnant women were fully informed about the study objectives and observation procedure. Information on the mothers’ current pregnancies was obtained through observation and their ANC card. Information on previous visits and their last children was collected from ANC cards. During the study, almost all of the women (98%) brought their ANC cards when visiting the ANC. Women who did not bring their cards were excluded from the study. The time spent for each ANC session, excluding waiting time and registration, was recorded.

Each observer noted the health provider’s performances during observation and then recorded the relevant information on the checklist, in addition to the information from the woman’s ANC cards and the time spent (in minutes) on the ANC consultation. At the end of each day, characteristics of the health providers (age, sex, profession and working experience) were collected in a brief interview. The observers sat approximately two meters away from the health providers and pregnant women to observe the consultation session.

### Data analysis

Data were entered into Microsoft Excel (2011) for Mac. Data were then transferred to ‘IBM SPSS 25’ for analysis. Descriptive statistics, such as percentage, mean (SD) and median (range), were applied to describe the characteristics of health providers and pregnant women’s personal information. The performance scores of health providers on history taking, physical examination, health promotion during pregnancy, for childbirth and postpartum care, including communication skills, were calculated as well as the time spent for each ANC session. When any performance item was conducted by the health provider regardless of whether it was right or wrong, a score of 1 was given; if the item was not done the score was 0.

Comparison of proportion between two groups was made using Chi-square or Fisher’s exact tests as appropriate. *Mann Whitney-U* or Student’s *t* tests were applied to compare medians or means between groups. The relationship between ANC performance scores of health providers and their working experience and age was explored using *Pearson* or *Spearman* correlation tests as appropriate, and multiple linear regression was applied to check for confounding by time-spent, age and working experience of health care providers.

## Results

A total of 77 ANC providers (20 from urban and 57 from rural areas) were enrolled in the study. During the study period, 421 observation sessions with 421 pregnant women (81 in urban and 340 in rural areas) were conducted.

The characteristics of health providers are shown in Table 2. Their overall mean age was 35 years, 98.7% were female and about 60% were nurses. Their median working experience was 11 years, ranging from 1 to 33 years, and was significantly higher in urban [17 (1–33)] than in rural [9 (1–29)] areas (*P* = 0.042).

**Table 2:** Characteristics of ANC health care providers*

Variable	Total (*N* = 77)^a^	Urban (*n* = 20)	Rural (*n* = 57)	*P*-value
Age (years)				
20–30	28 (36.8%)	6 (30.0%)	22 (38.6%)	
31–40	24 (31.2%)	4 (20.0%)	20 (35.1%)	0.139
41–52	25 (32.5%)	10 (50.0%)	15 (26.0%)	
Mean (SD)	34.95 (8.76)	38.20 (9.64)	33.81 (8.09)	0.051
Sex				
Female	76 (98.7%)	20 (100.0%)	56 (98.2%)	
Male	1 (1.3%)	0 (0.0%)	1 (1.8%)	1.00
Professional				
General practitioner	14 (18.2%)	5 (25.0%)	9 (15.8%)	
Nurse	46 (59.7%)	10 (50.0%)	36 (63.2%)	0.542
Midwife	17 (22.1%)	5 (25.0%)	12 (21.1%)	
Working experience (years)				
1–10	38 (49.4%)	8 (40.0%)	30 (52.6%)	
11–20	21 (27.3%)	4 (20.0%)	17 (29.8%)	0.123
21–33	18 (23.4%)	8 (40.0%)	10 (17.5%)	
Median (range)	11 (1–33)	17 (1–33)	9 (1–29)	0.042

^a^This is the number of observations recorded from 77 health providers who provided ANC

*Data shown as number (%) unless indicated

The characteristics of pregnant women who visited ANC are presented in Table 3. These women were relatively young, with mean age of 25 years, which was not significantly different in urban and rural areas. At the time of observation, 55.6% of the pregnant women were visiting ANC in their third trimester; the overall median and range of gestational age was similar in urban and rural areas. Approximately one third of the women came for ANC alone, although this was significantly more common in urban than in rural areas (*P* = 0.003).

**Table 3:** Characteristics of pregnant women who visited ANC*

Variable	Total (*N* = 421)^a^	Urban (*n* = 81)	Rural (*n* = 340)	*P*-value
Age of pregnant woman (years)				
14–17	26 (6.6%)	2 (2.7%)	24 (7.5%)	0.281
18–24	178 (44.9%)	37 (49.3%)	141 (43.9%)	
25–45	192 (48.5%)	36 (48.0%)	156 (48.6%)	
Mean (SD)	25.02 (5.88)	24.95 (5.02)	25.04 (6.07)	0.825
Number of pregnancies				
Median (range)	2 (1–7)	2 (1–7)	2 (1–7)	0.398
Gestational age (weeks):				
First trimester (<=12)	31 (7.8%)	4 (5.3%)	27 (8.4%)	
Second trimester (13–24)	145 (36.6%)	29 (38.7%)	116 (36.1%)	
Third trimester (25–41)	220 (55.6%)	42 (56.0%)	178 (55.5%)	0.655
Median (range)	26 (9–41)	25 (11–41)	26 (9–41)	0.807
Number of live births				
None	165 (39.2%)	26 (32.1%)	139 (40.9%)	0.146
More than one live birth	256 (60.8%)	55 (67.9%)	201 (59.1%)	
Median (range)	1 (0–6)	1 (0–6)	1 (0–6)	0.333
Accompanied or not				
Alone	163 (38.7%)	43 (53.1%)	120 (35.3%)	0.003
With others	258 (61.3%)	38 (46.9%)	220 (64.7%)	

^a^This is the number of observations recorded from 421 pregnant women who visited ANC

*Data shown as number (%) unless indicated

Overall, the medical history taking and physical examination performance by the health providers were relatively poor, as shown in Table 4. Six questions were related to ‘medical history taken’. Out of a maximum possible score of 6, the median was 3, and it was significantly higher in rural than in urban areas (*P* = 0.007). Half of the providers asked about the mothers’ last menstruation, prior pregnancies, current pregnancy problems and any medicines taken by the mothers during their pregnancies, while only one third of them asked about mother’s previous pregnancy problems. The percentage of the providers who asked about mothers’ age, last menstruation, and prior pregnancies was significantly lower in urban as compared to rural areas (*P* < 0.001, *P =* 0.01, and *P* = 0.002, respectively). The median score out of 6 was significantly lower in urban than in rural areas (*P* = 0.007). Observation of performance of the six items of physical examination resulted in higher scores, which were significantly lower in rural than in urban areas (*P* = 0.002) (Table 3). However, looking at the average scores, it appears that only examination of the abdomen was done in more than 70% of the cases, while in only 41.6% of the consultations did the health providers listen to the fetal heartbeat.

**Table 4:** Mothers’ medical history taking and physical examination by health providers*

Variable	Total (*N* = 421)^a^	Urban (*n* = 81)	Rural (*n* = 340)	*P*-value
Asking age of mothers				
yes	272 (64.6%)	19 (23.5%)	253 (71.4%)	< 0.001
Asking medication taken				
yes	232 (55.1%)	42 (51.9%)	190 (55.9%)	0.521
Asking last menstruation				
yes	210 (49.9%)	30 (37.0%)	180 (52.9%)	0.010
Asking prior pregnancy				
yes	250 (59.4%)	36 (44.4%)	214 (62.9%)	0.002
Asking previous pregnancy problem				
yes	139 (33.0%)	30 (37.0%)	109 (32.1%)	0.329
Asking current pregnancy problem				
yes	216 (51.3%)	48 (59.3%)	168 (49.4%)	0.111
Total score of history taking (max 6)				
Median (range)	3 (0–6)	2 (0–6)	3 (0–6)	0.007
Measuring weight and height				
yes	293 (69.6%)	57 (70.4%)	236 (69.4%)	0.866
Recording weight and fetus growth in the follow up book				
yes	287 (68.2%)	61 (75.3%)	226 (66.5%)	0.12
Taking blood pressure				
yes	260 (61.8%)	62 (76.5%)	198 (58.2%)	0.002
Examining edema				
yes	272 (64.6%)	66 (81.5%)	206 (60.6%)	< 0.001
Examining abdomen				
yes	311 (73.9%)	58 (71.6%)	253 (74.4%)	0.605
Listening to heart beat of fetus				
yes	175 (41.6%)	55 (67.9%)	120 (53.3%)	< 0.001
Score of physical examination (max 6)				
Median (range)	5 (0–6)	5 (0–6)	4.5 (0–6)	0.002
Washing hands before examination				
yes	28 (6.7%)	1 (1.2%)	27 (7.9%)	0.017
Asking permission any time before performing examination				
yes	86 (20.4%)	58 (71.6%)	28 (8.2%)	< 0.001
Giving feedback on the results of examination to the mothers				
yes	128 (43.2%)	59 (72.8%)	123 (36.2%)	< 0.001
Considering privacy during examination				
yes	17 (4.0%)	10 (12.3%)	7 (2.1%)	< 0.001
Score of additional procedures (max 4)				
Median (range)	1 (0–3)	2 (0–3)	0 (0–3)	< 0.001
Grand total score of all physical examination performances (max 10)				
Median (range)	5 (0–9)	6 (1–9)	5 (0–9)	< 0.001

^a^This is the number of observations recorded from 421 pregnant women who visited ANC

*Data shown as number (%) unless indicated

The providers’ performance on other procedures was also poor. For example, washing hands before examination was only 6.7% and privacy during examination was ensured in only 4.0% of cases. The grand total score on the physical examination process was significantly higher in urban than in rural areas (*P* < 0.001).

Although the health providers from urban areas provided more health promotion messages to the mothers as compared to those in the rural areas, their overall performance for this service remained unsatisfactory (Table 5). For example, each of eight items separately was performed in less than 50% of the observations. The overall median score out of a possible 8 on health promotion during pregnancy was only 2 and was significantly lower in rural than urban areas (*P* < 0.001).

**Table 5:** Provision of health promotion during pregnancy, and on childbirth and postpartum care to mothers by health providers*

Variable	Total (*N* = 421)^a^	Urban (*n* = 81)	Rural (*n* = 340)	*P*-value
Explaining disease prevention				
yes	88 (20.9%)	46 (56.8%)	42 (12.4%)	< 0.001
Providing information on how to seek care if danger sign is observed				
yes	171 (40.6%)	54 (66.7%)	117 (34.4%)	< 0.001
Explaining on physical activities to be done during pregnancy				
yes	121 (28.7%)	47 (58.0%)	74 (21.8%)	< 0.001
Advising pregnant women to take more rest than usual				
yes	214 (50.8%)	53 (65.4%)	161 (47.4%)	0.003
Advising mothers not to restrict food				
yes	168 (39.9%)	55 (67.9%)	113 (33.2%)	< 0.001
Advising mothers not to drink nor smoke				
yes	118 (28.0%)	34 (42.0%)	84 (24.7%)	0.002
Advising mothers to eat variety of food				
yes	71 (16.9%)	45 (55.6%)	26 (7.6%)	< 0.001
Giving iron/folate/vitamin to mothers				
yes	105 (24.9%)	45 (55.6%)	60 (17.6%)	< 0.001
Total score of health promotion during pregnancy over 8, Median (range)	2 (0–8)	5 (0–8)	2 (0–6)	< 0.001
Advising a safe place for childbirth				
yes	199 (47.3%)	44 (54.3%)	155 (45.6%)	0.157
Providing information on signs of labour				
yes	180 (42.8%)	46 (56.8%)	134 (39.4%)	0.004
Providing childbirth preparedness				
yes	167 (39.7%)	38 (46.9%)	129 (37.9%)	0.138
Advising on postpartum care at health facilities				
yes	48 (11.4%)	17 (21.0%)	31 (9.1%)	0.003
Advising to bring the child for vaccination				
yes	65 (15.4%)	17 (21.0%)	48 (14.1%)	0.124
Providing child vaccination counseling				
yes	0	0	0	
Providing breastfeeding counseling				
yes	0	0	0	
Providing nutrition counseling				
yes	0	0	0	
Advising postpartum mothers to eat a variety of food				
Yes	42 (10.0%)	30 (37.0%)	12 (3.5%)	< 0.001
Informing on diet habits to improve malnutrition				
yes	50 (11.9%)	14 (17.3%)	36 (10.6%)	0.094
Informing on complementary food for child at 6 to 23 months				
Yes	33 (7.8%)	21 (25.9%)	12 (3.5%)	< 0.001
Suggesting one/two options to improve nutrition status				
Yes	53 (12.6%)	26 (32.1%)	27 (7.9%)	< 0.001
Total scores of health promotion for childbirth and postpartum period over 12				
Median (range)	2 (0–7)	3 (0–8)	2 (0–7)	< 0.001

^a^This is the number of observations recorded from 421 pregnant women who visited ANC

*Data shown as number (%) unless indicated

Health promotion on childbirth and postpartum care to mothers by the providers both in urban and rural areas was also very poor, as illustrated by the data in Table 4. For example, just 47.3% of the providers gave advice on a safe place for childbirth, and only 15% advised mothers to bring their children for vaccination. None of them provided counseling on child vaccination, breastfeeding and nutrition. The overall median score for health promotion on childbirth and the postpartum period out of a possible 12 was only 2; although both were very low, the score was significantly higher in urban than in rural areas (*P* < 0.001).

The availability of health education materials for each ANC session observed and their use during health education by health providers at the public health facilities was highly inadequate as shown in Table 6. Health education materials were used to provide health information in fewer than 10% of the sessions, although more than 50% of required health education materials were available at each health facility at the time. The very low frequency of using materials to explain about diet during pregnancy (9.7%), after childbirth (2.6%), and for child vaccination (3.6%) was not significantly different between urban and rural areas.

**Table 6:** Health education materials used by health providers at health facilities*

Variable	Total (*N* = 421)^a^	Urban (*n* = 81)	Rural (340)	*P*-value
Using any health education material to explain diet during pregnancy				
Yes	40 (9.5%)	3 (3.7%)	37 (10.9%)	0.056
Using any health education material to explain diet after childbirth				
Yes	11 (2.6%)	1 (1.2%)	10 (2.9%)	0.699
Using any health education material to explain about vaccination				
Yes	15 (3.6%)	3 (3.7%)	12 (3.5%)	1.000
Availability of materials for each ANC session at clinics				
Yes	230 (54.6%)	43 (53.1%)	187 (55.0%)	0.756

^a^This is the number of observations recorded from 421 pregnant women who visited ANC

*Data shown as number (%) unless indicated

The results of assessing the performance of health providers’ communication with the mothers are presented in Table 7. Generally, the communication between health providers and their clients had a lot of weaknesses. Except for sitting at the same level and using simple language with the mothers, other communication items expressed or performed by the health providers were inadequate. For example, the items helping brainstorming, agreeing on an appointment for the next visit, asking if there are more questions, and encouraging to ask questions were almost never performed. The overall median score out of 24 on communication was only 6; there was no significant difference between urban and rural areas.

**Table 7:** Communication, behaviour and attitude of health providers*

Variable	Total (*N* = 421)^a^	Urban (*n* = 81)	Rural (340)	*P*-value
Warmly greeting				
Yes	97 (23.0%)	42 (51.9%)	55 (16.2%)	< 0.001
Self introduction with title				
Yes	50 (11.9%)	4 (4.9%)	46 (13.5%)	0.032
Sitting at the same level as mothers				
Yes	390 (92.6%)	80 (98.8%)	310 (91.2%)	0.019
Paying attention to mothers				
Yes	144 (34.2%)	21 (25.9%)	123 (36.4%)	0.081
Listening carefully to mothers				
Yes	143 (34.0%)	27 (33.3%)	116 (34.1%)	0.893
Kindness				
Yes	103 (24.5%)	15 (18.5%)	88 (25.9%)	0.166
Confidentiality with conversation				
Yes	120 (28.5%)	16 (19.8%)	104 (30.6%)	0.052
Considering privacy				
Yes	52 (12.4%)	18 (22.2%)	34 (10.0%)	0.003
Asking open questions				
Yes	153 (36.3%)	28 (34.6%)	125 (36.8%)	0.712
Giving practical messages				
yes	99 (23.5%0	20 (24.7%)	79 (23.2%)	0.781
Giving brief and relevant information				
yes	267 (63.4%)	61 (75.3%)	206 (60.6%)	0.013
Using body language				
yes	165 (39.2%)	38 (46.9%)	127 (37.4%)	0.113
Using simple language				
yes	369 (87.6%)	56 (69.1%)	313 (92.1%)	< 0.001
Avoiding judging words				
yes	136 (32.3%)	24 (29.6%)	112 (32.9%)	0.567
Giving more time to think				
yes	85 (20.2%)	25 (30.9%)	60 (17.6%)	0.008
Helping with brainstorming				
yes	4 (1.0%)	0 (0.0%)	4 (1.2%)	1.000
Encouraging to ask questions				
yes	19 (4.5%)	6 (7.4%)	13 (3.8%)	0.163
Recognizing good practice				
yes	71 (16.9%)	16 (19.8%)	55 (16.2%)	0.440
Reflecting/repeating the words of women				
yes	30 (7.1%)	8 (9.9%)	22 (6.5%)	0.284
Showing compassion to the issues				
yes	106 (25.2%)	13 (16.0%)	93 (27.4%)	0.035
Asking for more questions				
yes	15 (3.6%)	4 (4.9%)	11 (3.2%)	0.502
Agreeing on an appointment for next visit				
yes	8 (1.9%)	2 (2.5%)	6 (1.8%)	0.653
Practicing two way-communication				
yes	13 (3.1%)	4 (4.9%)	9 (2.6%)	0.287
Thanking, respecting, and showing good manners at the end of session				
yes	84 (20.0%)	15 (18.5%)	69 (20.3%)	0.719
Total scores of communication scores (max 24)				
Median (Range)	6 (1–18)	6 (1–18)	6 (1–18)	0.919

^a^This is the number of observations recorded from 421 pregnant women who visited ANC

^*^Data shown as number (%) unless indicated

The overall mean time spent for ANC per visitor was 16.21 min (SD 4.28), and it was significantly longer in rural [20.65 (4.92)] than in urban [15.15 (3.33)] areas (*P* < 0.001). Paradoxically, significant negative correlations were found between time spent and physical examination scores [*r* = − 1.117*, *P* = 0.016] and time spent and health promotion [*r* = − 1.200*, *p* = 0.013]. Time spent for medical history scores and counseling scores were not correlated. Total performance scores were also not significantly correlated, though a negative trend was observed.

Further analysis using Pearson or Spearman correlation tests revealed correlations between health providers’ working experience and age and their performance scores. There was a significantly positive correlation between working experience and age of health providers and their physical examination performance scores [*r* = 0.301** (*n* = 77), *P* = 0.008 and *r* = 0.285** (*n* = 77), *P* = 0.024, respectively]. In a multiple linear regression analysis, working experience of health providers was independently correlated with their physical performance scores (adjusted R square = 0.017).

## Discussion

To the best of our knowledge, this is the first study conducted in Laos to assess the working performance of the ANC health workers, with special emphasis on the provision of information and counseling to their clients by using structured observation at public health facilities. The findings demonstrate that the overall performance of ANC providers was very poor in both urban and rural areas.

Thus although women are accessing ANC, they are not benefiting optimally from ANC services. They are not sufficiently protected through direct screening; do not get enough information on safe pregnancy or on how to nurture the child in the first 1000 days. Medical history taking and physical examination in ANC service are crucial for initial assessment to detect danger signs that may need treatment or referral to a higher level health facility [6, 12]. We found that performance on history taking remained poor, while physical examination was adequate but needs improvement. It is especially worrisome that in fewer than half of the cases observed did providers listen to the fetal heartbeat. Similar findings were reported from Tanzania [25]. If ANC is not carefully conducted, preventable health issues can be missed, such as hypertension and edema (pre-eclampsia and eclampsia), of caus important maternal death during pregnancy [26].

We found that medical history taking by health providers was better in rural than in urban areas, but the opposite was true for physical examination and other procedures. There may be different reasons for this finding. For example, time constraints can be an important factor, contributing to inadequate performance by health providers where facilities are overcrowded (submitted for publication). In this study, however, there was a negative correlation between time spent per consultation and performance in physical examination and health promotion. The approximate number of clients per day was 40 in urban and 10 in rural areas. This finding therefore requires further research. On the other hand, ANC services in urban areas often focus on high-risk screening in addition to routine ANC, while the rural areas provide basic services.

The results of our study clearly demonstrate that the provision of health promotion messages by health workers to clients in both urban and rural areas remains inadequate. During ANC visits, all pregnant women should have the opportunity to be fully informed about their pregnancy and given evidence-based information to help them make decisions [2, 6, 12]. Unfortunately, the pregnant women in the locations we studied received very little information; less than one quarter of them was given health promotion messages. The situation was similar situation in rural Burkina Faso, Uganda and Tanzania, where less than 20% of health workers provided health education to pregnant women and only 2 to 7% of women received information on danger signs of pregnancies [27].

Strengthening the relationship between pregnant women, their family members and health providers would also help to promote childbirth at health facilities, as well as breastfeeding and a healthy lifestyle [12, 18]. Unfortunately, family members who accompanied pregnant women to visit ANC in our study did not have the opportunity to be informed about care, treatment, and or possibility of unexpected problems, because they were not invited to attend during ANC sessions together with pregnant women (submitted for publication).

Although effective counseling can help to improve women’s behavior and health outcomes [15, 16], none of the health providers in this study performed counseling on nutrition, child vaccination, and breastfeeding. This was probably due to lack of training, guidelines, and a specific private room for counseling in the public health facilities in Laos [28, 29].

Information materials seemed to be a neglected component for ANC provision at public health facilities in Laos. Our study demonstrated that ANC health care providers rarely apply IEC materials for their health promotion activities even when they were available – this may be due to insufficient materials and/or to lack of good training. This is supported by the findings from another recent study in which health workers reported that they lacked materials and guidelines to use at their health facilities, and a lack of training on how to use existing materials effectively (submitted for publication).

The health care providers’ communication skills influence women’s satisfaction with maternal health care services [30]. Improvement of those communication skills, for example by training, should enhance their information provision and increase women’s involvement in their own health care [31]. The results of this study also suggest that communication between health care providers and their clients is still very weak. Health care providers need to show more kindness and respect, and need more privacy for communication with their clients. A study in Ethiopia on clients’ satisfaction with health care providers’ communication also demonstrated that pregnant women were particularly dissatisfied about information provision and the time limitations of consultations [32].

### Limitations of the study

The purposive sampling method used for the study meant that the findings may not be widely generalizable. Not all behaviors of the health care providers are open to observation and the observers may have personal bias. There was no video recording, so important information during the observation might have been missed. The observation itself might influence actual practices between health care providers and clients. Comparative analysis between over and covert observation methods is recommended for further observational study.

## Conclusion

The overall performance of ANC services by health care providers at the public health facilities in Lao PDR was observed to be inadequate in both urban and rural areas. Insufficient provision of health education and poor communication skills of health care providers were revealed. Existing IEC materials were scarcely used for health education by health care providers both in urban and rural areas even when they were available. Taking action to improve the quality of ANC services by training and providing specific guidelines, creating separate rooms, and providing sufficient and effective materials to be used for counseling is urgently required at the public health facilities in Laos.

## Data Availability

Authors would like to confirm that the raw data of the manuscript can be provided with an appropriate request.

## Abbreviations

ANC: Ante-natal Care
IEC: Information Education and Communication
Lao PDR: Lao People’s Democratic Republic
LMIC: Low- and Middle-Income Countries
MCNV: Medical Council Netherland and Vietnam
WHO: World Health Organization
